# The Role of MUC1 in Chronic Rhinosinusitis with Nasal Polyps (CRSwNP): The Correlation with Disease Severity

**DOI:** 10.3390/jpm15110547

**Published:** 2025-11-10

**Authors:** Rossana Giancaspro, Giuseppe Stefano Netti, Federica De Luca, Valentina Camporeale, Valeria Catalano, Michele Cassano, Elena Ranieri, Matteo Gelardi

**Affiliations:** 1 Unit of Otolaryngology, Department of Clinical and Experimental Medicine, University of Foggia, Via Luigi Pinto 1, 71122 Foggia, Italy; michele.cassano@unifg.it (M.C.); matteo.gelardi@unifg.it (M.G.); 2 Unit of Clinical Pathology, Center for Molecular Medicine, Department of Medical and Surgical Sciences, University of Foggia, 71122 Foggia, Italy; giuseppestefano.netti@unifg.it (G.S.N.); federica.deluca@unifg.it (F.D.L.); valentina.camporeale@unifg.it (V.C.); valeria.catalano@unifg.it (V.C.); elena.ranieri@unifg.it (E.R.)

**Keywords:** chronic rhinosinusitis with nasal polyps, MUC-1, mucins, clinical cytological grading, eosinophils, mast cells

## Abstract

**Background:** Mucins, particularly MUC1, are involved in the pathogenesis of chronic respiratory diseases. In chronic rhinosinusitis with nasal polyposis (CRSwNP), altered mucin expression may contribute to chronic inflammation and tissue remodeling. However, the specific role of MUC1 in CRSwNP and its correlation with clinical severity and inflammatory pathway remains unclear. **Objective:** We aimed to evaluate the MUC-1 expression in nasal polyps of patients with CRSwNP and to assess the correlation of MUC-1 expression and disease severity, according to Clinical-Cytological Grading (CCG). **Methods:** Eighteen consecutive patients with CRSwNP who underwent endoscopic sinus surgery (ESS) were enrolled. A double-label immunofluorescence was performed to evaluate the expression of MUC-1, CD15 and Tryptase and their eventual co-localization on histological samples. Double-positive MUC-1+CD15+ and MUC-1+Tryptase+ inflammatory cells were counted by confocal microscopy. **Results:** MUC1 was expressed in all samples, with a significantly increasing expression in relation to CCG (*p* < 0.001). A significant co-localization between MUC1 and CD15+ eosinophils was observed, with a progressive increase in the number of double-positive cells from low to high CCG (*p* < 0.001). On the contrary, the co-localization between MUC1 and Tryptase+ mast cells was not significant, although both markers showed a higher expression in cases with high CCG (*p* < 0.001). **Conclusions:** A strong correlation between CRSwNP severity and MUC-1 expression, mainly colocalized with infiltrating eosinophils, was shown. This offers a promising perspective for the use of MUC-1 as a biomarker of CRSwNP.

## 1. Introduction

In recent years, the study of mucins has gained significant attention due to their critical role in the pathophysiology of chronic respiratory diseases, including asthma, Chronic Obstructive Pulmonary Disease (COPD) and CRSwNP. Mucins, primarily composed of glycoproteins, are key structural components of mucus, which serves as the first line of defense against pathogens and harmful environmental agents in the airway. In CRSwNP, the overproduction and altered composition of mucins are associated with the chronic inflammation and tissue remodeling characteristic of the disease. Specifically, mucins such as MUC1, MUC5AC, and MUC5B are thought to play important roles in the regulation of the immune response and in the maintenance of airway epithelial integrity [1,2].

The understanding of mucin expression in CRSwNP, particularly in relation to disease severity, is still evolving. While MUC1 has been traditionally described as an anti-inflammatory molecule in various airway diseases, emerging evidence suggests that its role might be more complex. Some studies have shown the involvement of MUC1 in promoting inflammation, especially in the context of eosinophilic inflammation, which is prevalent in Type 2 CRSwNP [3]. Furthermore, MUC1 has been implicated in regulating the survival of eosinophils, a key feature in the inflammatory response in CRSwNP [2]. Given its possible dual role, the relationship between MUC1 expression and disease severity in CRSwNP remains unclear, necessitating further investigation.

Based on this background, the current study aimed to investigate the expression of MUC1 in nasal polyps, its correlation with disease severity as assessed by Clinical Cytological Grading (CCG), and its interaction with other inflammatory cells, such as eosinophils and mast cells.

## 2. Materials and Methods

Eighteen consecutive patients, of whom 13 were males (72.22%), who underwent endoscopic sinus surgery (EES) for chronic rhinosinusitis with nasal polyposis (CRSwNP) at the Department of Otolaryngology, University Hospital of Foggia, were included in the study. Diagnosis of CRSwNP was based on EPOS2020 criteria for symptoms and endoscopic and sinus CT findings [4].

The patients’ ages ranged from 30 to 74 years, with a median age of 54.5 years. One week before surgery, each patient underwent a thorough clinical and anamnestic evaluation, as well as nasal cytology and nasal endoscopy. During surgery, under general anesthesia, endoscopically guided biopsy samples from nasal polyps were collected using Weil-Blakesley forces.

Specific exclusion criteria were the presence of acute or chronic upper respiratory infections, previous or current specific immunotherapy, and use of nasal or oral corticosteroids, nasal or oral vasoconstrictors, antileukotrienes, and antihistamines during the previous 6 weeks.

All participants provided written informed consent. The study was approved by the Area 1 Ethics Committee of the University Hospital of Foggia (44/CE/2022 dated 4 April 2022, and DCS n. 131 dated 19 April 2022).

### 2.1. Medical History

Each patient’s clinical history was carefully assessed, with a specific focus on atopy (including total IgE levels and skin prick tests for inhalant allergens), asthma, asthma, aspirin and other nonsteroidal anti-inflammatory drugs sensitivity (NSAIDs), as well as the number of previous polypectomy procedures.

Moreover, in order to assess patients’ health-related quality of life (HRQoL), they were asked to fill out the Sino-Nasal Outcome Test (SNOT-22), a disease-specific questionnaire designed to assess five distinct domains, including rhinologic symptoms, extra-nasal rhinologic symptoms, ear/facial symptoms, psychological dysfunction, and sleep dysfunction [5,6].

### 2.2. Nasal Endoscopy

Preoperative nasal endoscopy was carried out by a 3.4 mm diameter flexible fiberscope (Vision-Sciences^®^ ENT-2000, Orangeburg, NY, USA) to confirm the diagnosis of CRSwNP. The size of sinonasal polyps was assessed using Meltzer endoscopic polyp scores (Total Nasal Polyp Score, TNPS) [7]. Based on this classification, each nasal cavity is scored from 0 to 4, with 0 indicating no visible nasal polyps and 4 indicating complete obstruction of the nasal cavity by nasal polyps. Combined left and right scores give a total possible score range of 0–8, with higher scores indicating larger nasal polyps and greater disease severity [8].

### 2.3. Nasal Cytology and Clinical-Cytological Grading

Cytological samples were preoperatively obtained using Nasal Scraping^®^ (EP Medica, Fusignano, Italy) and subsequently processed following validated criteria [9]. The predominant inflammatory cell type in each sample was considered, allowing classification into four cytologic phenotypes: neutrophilic, eosinophilic, mast-cell, and mixed cellularity (eosinophils and mast cells).

According to nasal cytology findings and comorbidities (asthma, nonsteroidal anti-inflammatory drug (NSAID)-exacerbated respiratory disease (NERD) and allergy), a specific Clinical-Cytological Grading (CCG) score was assigned to each patient.

Based on the obtained CCG score, the entire sample was subsequently divided into three groups corresponding to low, moderate, and high grades of severity.

### 2.4. Indirect Immunofluorescence and Confocal Laser Scanning Microscopy

Histological specimens from the 18 enrolled patients were processed at the Clinical Pathology Unit and the Center for Molecular Medicine of our institution.

From formalin-fixed and paraffin-embedded tissue blocks, serial sections of 4 μm thickness were prepared. The sections were deparaffinized in xylene, rehydrated through a descending ethanol series, rinsed for 5 min in distilled water, and mounted on slides coated with poly-L-lysine.

To examine the distribution and potential co-localization of MUC1, CD15, and tryptase, a dual immunofluorescence staining protocol was carried out. Primary antibodies included: rabbit polyclonal IgG anti-MUC1 (clone EPR11197; Sigma-Aldrich, Darmstadt, Germany); mouse monoclonal IgM anti-CD15 (clone MMA; Ventana Medical Systems, Tucson, AZ, USA), which recognizes CD15 expressed on most granulocytes, including eosinophils and neutrophils, and to a lesser extent on monocytes; and mouse monoclonal IgG1 anti-tryptase (clone G3; Ventana Medical Systems, Tucson, AZ, USA), a marker specific for mast cells.

After deparaffinization, the tissue sections were permeabilized using 0.2% Triton X-100, blocked with a mixture of 0.05% fetal bovine serum (FBS) and 2% bovine serum albumin (BSA), and incubated overnight at 4 °C with the antibody cocktail diluted in the same blocking buffer. Antigen–antibody complexes were visualized with the appropriate secondary antibodies: Alexa Fluor 488 goat anti-rabbit IgG, Alexa Fluor 546 goat anti-mouse IgM, and Alexa Fluor 546 goat anti-mouse IgG1 (Thermo Fisher Scientific, Waltham, MA, USA). Following three washes in PBS (5 min each), both samples and negative controls were exposed for 1 h at room temperature to the secondary antibodies diluted 1:250 in PBS. Nuclei were counterstained with TO-PRO (1:3000 in PBS, pH 7.4; Invitrogen–Molecular Probes, Thermo Fisher). The slides were then mounted with Gel Mount (Sigma-Aldrich) and sealed. Fluorescence imaging was carried out using a Leica TCS SP5 confocal microscope (Leica Microsystems, Wetzlar, Germany) equipped with argon–krypton (488 nm), green–neon (543 nm), and helium–neon (633 nm) lasers. Quantification of fluorescence intensity was performed following standardized image-analysis procedures described in previous research [10].

Inflammatory cells positive for MUC1, CD15, or tryptase were enumerated by two independent pathologists across ten randomly selected high-power fields (HPFs) per specimen.

## 3. Results

The study population was subdivided into three groups based on the CCG of CRSwNP: low (n = 6), medium (n = 6), and high (n = 6). The demographic characteristics, comorbidities, nasal cytology, CCG, as well as SNOT-22 and TNPS scores of each patient are summarized in Table 1.

**Figure 1:**
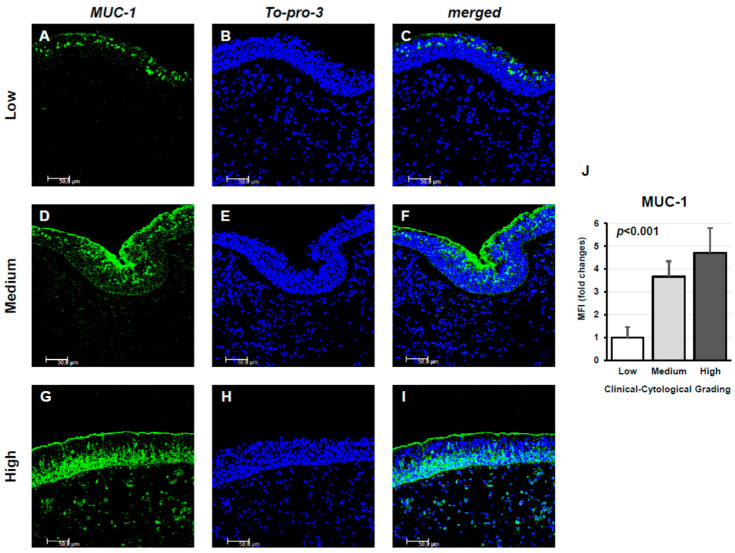
Immunofluorescence analysis of MUC1 expression in nasal tissue samples from CRSwNP patients stratified by Clinical–Cytological Grading (CCG). Panels (**A**–**I**) display MUC1 staining (green) in specimens with low (**A**–**C**), moderate (**D**–**F**), and high (**G**–**I**) CCG scores. Nuclear staining was achieved with To-Pro-3 (blue). Each panel represents one of six independent samples per severity group. Scale bar: 50 μm; magnification: 400×. Panel (**J**) illustrates the quantitative comparison of mean fluorescence intensity (MFI) for MUC1 among the three CCG categories, demonstrating a progressive increase in signal intensity with disease severity (ANOVA, *p* < 0.001).

Confocal microscopy analysis showed MUC-1 expression in tissue samples from the three study groups. MUC-1 was detectable in all examined tissues from CRSwNP patients. In the Low panels, characterized by a low CCG, MUC-1 expression was limited. In the Medium panels, a significant increase in MUC-1 expression was observed. Finally, in the High panels, MUC-1 expression was high, showing strong fluorescence and uniform distribution, with an elevated number of MUC-1 highly positive cells (Figure 1A–I). This result was corroborated by the measurement of specific fluorescence levels (*p* < 0.001; Figure 1J).

**Figure 2:**
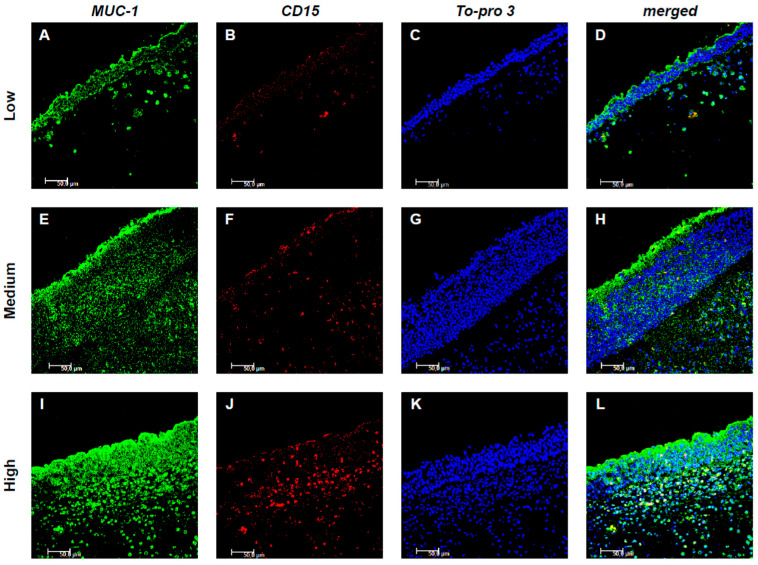
Colocalization of MUC-1 and CD15+ eosinophils. Panels (**A**–**L**) show double-label immunofluorescence of nasal mucosa from patients with low (**A**–**D**), medium (**E**–**H**), and high (**I**–**L**) CCG CRSwNP. MUC-1 is labeled in green, CD15 in red, and nuclei are counterstained with To-pro-3 (blue). In the merged images, yellow indicates colocalization, revealing that MUC-1 expression in eosinophils increases with higher CCG levels. These results represent six patients per group. Images were captured at 400× magnification; scale bar = 50 μm.

**Figure 3:**
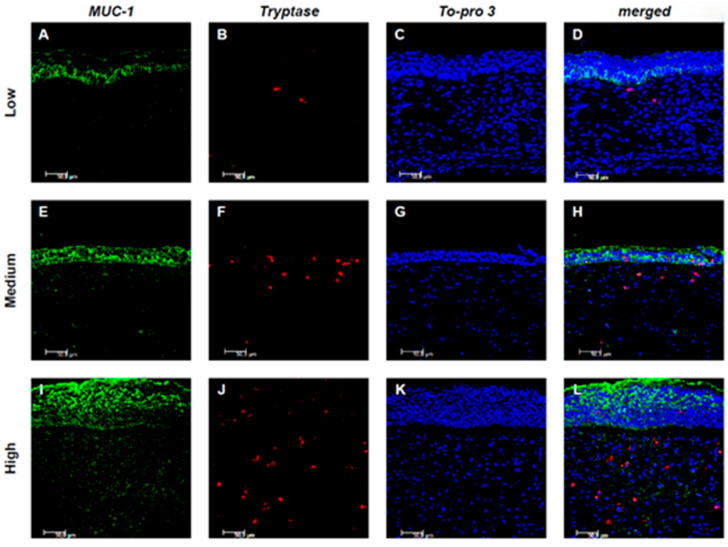
Colocalization of MUC-1 and Tryptase+ mast cells. Panels (**A**–**L**) show double-label immunofluorescence of nasal mucosa from patients with low (**A**–**D**), medium (**E**–**H**), and high (**I**–**L**) CCG CRSwNP. MUC-1 is shown in green, Tryptase in red, and nuclei are counterstained with To-pro-3 (blue). In the merged images, yellow indicates colocalization, revealing that MUC-1 expression in mast cells increases with higher CCG levels. Results are representative of six patients per group. Images were captured at 400× magnification; scale bar = 50 μm.

The expression of MUC-1 in both resident and inflammatory cells was subsequently analyzed. Notably, MUC-1 showed a significant colocalization with CD15+ eosinophils (Figure 2), whereas no colocalization was observed with Tryptase+ mast cells (Figure 3). In particular, in low-GCC patients, a limited expression of both MUC-1 and CD15 was shown. On the contrary, the expression of MUC-1 and CD15 significantly increased as medium and high CCG tissues were examined, respectively. Furthermore, when assessing inflammatory CD15+ cells expressing MUC-1, their number progressively increased across tissues with low, medium, and high CCG grades. Quantitative analysis of double-positive MUC-1+/CD15+ eosinophils confirmed this trend, showing a significant rise in their count in relation to CCG severity (*p* < 0.001), suggesting a potential synergistic interaction between the two markers, related to the progression and severity of the disease (Figure 2A–L).

Evaluating the expression of MUC-1 and Tryptase, it was observed that in low CCG samples, the expression of both markers was limited or absent, with no significant co-localization between the two markers. In medium CCG samples, the expression of both markers increased, with limited co-localization. However, a statistically significant correlation between the expression of MUC-1 and Tryptase was found only in high CCG samples, although without colocalization, suggesting distinct but potentially related processes (Figure 3A–L).

**Figure 4:**
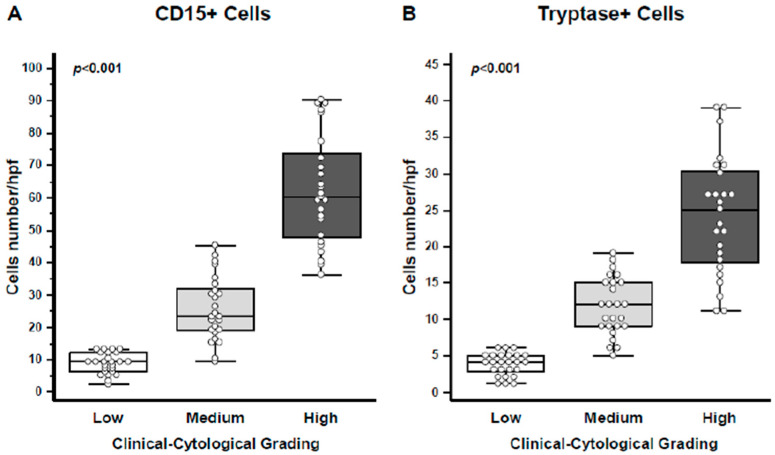
Quantification of eosinophils and mast cells. (**A**) CD15+ eosinophils were quantified, showing a progressive increase in cell numbers per high-power field (HPF) with higher CCG levels (ANOVA, *p* < 0.001). (**B**) Tryptase+ mast cells were similarly quantified, demonstrating an increase in cell numbers per HPF with rising CCG (ANOVA, *p* < 0.001). In the graphs, data are presented as medians with 25th and 75th percentiles shown as boxes, 5th and 95th percentiles as whiskers, and individual values indicated by circles.

A detailed quantification of both CD15+ eosinophils and Tryptase+ mast cells revealed a progressive increase in their numbers in correlation with the CCG score (*p* < 0.001 for both eosinophils and mast cells; Figure 4A,B).

## 4. Discussion

Airway mucin overproduction is nowadays considered the hallmark risk factor of several chronic respiratory diseases, including asthma, chronic obstructive pulmonary disease (COPD) and cystic fibrosis [11]. According to the unified airway theory, which suggests that the nose and lungs share common pathogenic mechanisms and structural changes, mucins play a critical role in the pathophysiological changes in CRS as well [12].

Since no effective targeted treatment for mucin overproduction in the airways has been developed to date, there is growing interest among researchers in developing personalized and effective treatments aimed at regulating mucin expression, which are the main components of mucus.

Mucus is the first-line defense barrier of the airway epithelium against several pathogens and environmental agents. In healthy individuals, mucociliary clearance (MCC) is part of the innate defense mechanism and consists of two equally important components: mucus production and mucus transport. When MCC is compromised, airways become vulnerable to a vicious cycle of infection and obstruction. This phenomenon is particularly evident in patients with CRS who experience relentless cycles of infection and inflammation, resulting in ciliary loss and hyper-viscous mucus blanket, which generates dysfunctional mucociliary coupling [13].

The sources of mucus secretion are goblet cells present in an outer epithelial layer and submucosal glands. The human respiratory mucus is mostly composed of water (95%). The solid phase is composed of mucins, non-mucin proteins, enzymes, salts, lipids, and cellular debris, which allows the mucus to be classified as a gel with properties of both a soft, elastic solid and a viscous fluid [14].

**Figure 5:**
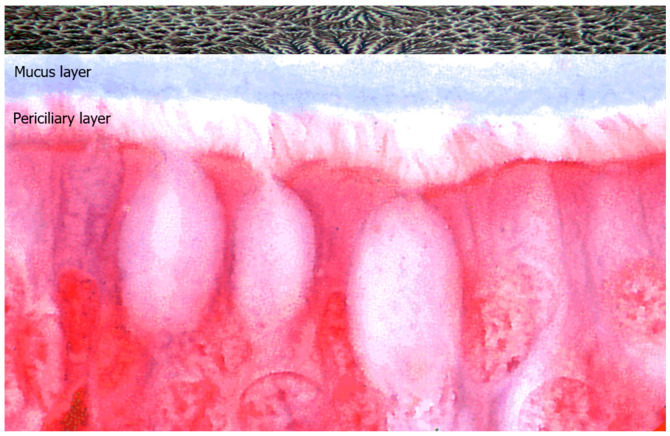
Representation of ciliated epithelial cells covered by two distinct mucin distribution layers: the mucus layer and the periciliary layer. Above, an example of nasal ferning in non-pathologic mucosa, observed under phase-contrast microscopy at 1000× magnification.

The hydration status of the mucus is measured as the wet-to-dry content of mucus or as the mucin concentration, which is the major component of mucus. The secreted mucin polymers interweave to form mesh-like gels with mesh sizes that are concentration dependent. Mucins are distributed in two different layers: the mucus layer and the periciliary layer. The periciliary layer is a dense gel composed of transmembrane or membrane-bound glycopolymers, including the MUC1, MUC3, MUC4, MUC12, MUC16, and MUC17 mucins, which are tethered to epithelial-cell surfaces and cilia. The mucus layer is composed of secreted/gel-forming mucins, such as MUC2, MUC5, MUC6, and MUC19. When there is a balance of non-aqueous components, the evaporation of water can lead to crystallization of the electrolytes, and the fern formation occurs. The fern pattern varies depending on the patient’s health status and can be evaluated according to Rolando’s score: healthy patients have a type I (uniform fern pattern with closely branching arborization) or type II (well-distinguished but less branching arborization) fern pattern. Patients suffering from rhinopaties present with “pathologic” type III (scarce or single ferns) or type IV (absence of ferns) fern patterns [15] (Figure 5).

Under physiological conditions, mucins serve various functions, including cell proliferation, inflammation, immune response, and cell–cell adhesion. However, abnormal mucin expression, decreased clearance, and excessive mucus formation can lead to airway obstruction and exacerbation of preexisting airway disease, and turn a powerful innate cleaning defense system into a detrimental mechanism [16].

Currently, research on mucin primarily focuses on MUC5AC and MUC5B. In particular, MUC5AC has been shown to play a key role in the inflammatory response of the respiratory tract in general, while MUC5B appears to have a specific role in the development of CRS [17,18]. Nevertheless, the chronic inflammatory pathways underlying CRS result in cellular alterations and structural remodeling of the nasal mucosa that lead to a change in the rheological properties of mucus, with altered expression of other mucins, including MUC1 [19].

MUC1 is a highly glycosylated transmembrane protein expressed mainly in the apical surface of most glandular epithelial cells. Interestingly, MUC1 is known to display a regulatory role in mucosal immunity and is primarily involved in the formation of a physical barrier to lubricate and protect normal epithelial tissues, limiting infection and colonization, and mediating signal transduction. Several studies have shown that MUC1 acts as an anti-inflammatory molecule in several airway infections and mediates the expression of anti-inflammatory genes in lung diseases such as chronic rhinosinusitis, chronic obstructive pulmonary disease, and severe asthma [20]. However, dysregulated and inappropriately glycosylated MUC1 have been correlated with chronic epithelial diseases and cancers, suggesting that the perturbation of the general function of this glycoprotein can alter tissue homeostasis [21].

Recent evidence also indicates that interleukin-22 (IL-22), a cytokine belonging to the IL-10 family, can modulate MUC1 expression in nasal epithelial cells. IL-22 and its receptor, IL-22R1, are both expressed in nasal polyps, mainly in inflammatory and epithelial cells, respectively. In vitro studies have demonstrated that stimulation with Staphylococcus aureus exotoxins induces IL-22 production, which in turn significantly enhances MUC1 mRNA expression in nasal polyp cells. The levels of IL-22-induced MUC1 expression were found to correlate positively with IL-22R1 expression, suggesting that the IL-22/IL-22R1 axis directly regulates epithelial MUC1 synthesis. This pathway might contribute to the epithelial response to chronic inflammation in CRSwNP, linking microbial stimulation, cytokine signaling, and mucin regulation. Moreover, the imbalance between IL-22 and IL-22R1 expression could influence local eosinophilia and disease severity through modulation of MUC1-dependent mechanisms [22].

Since the role of MUC1 in CRSwNP remains poorly understood, we decided to conduct an immunofluorescence study to evaluate MUC1 localization within nasal polyps’ samples and evaluate its potential correlation with disease severity.

As shown in the results, the tissue levels of MUC1 in nasal mucosa samples progressively increased across specimens with low, moderate, and high CCG grades, showing a correlation with the severity of CRSwNP. Interestingly, previous studies had shown the anti-inflammatory function of MUC1 in asthma, by reducing the NLRP3 inflammasome-mediated pyroptosis through the inhibition of the TLR4/MyD88/NF-κB pathway, thereby suppressing neutrophilic airway inflammation in patients with asthma [23]. Moreover, MUC1 downregulation was associated with TNF-α-induced necroptosis in human bronchial epithelial cells by mediating the RIPK1/RIPK3 pathway [24]. Precisely because MUC1 has traditionally been attributed anti-inflammatory properties both in the respiratory tract and in extrapulmonary tissues, so much so that it has been defined as a “peacemaker”, we hypothesized that it would inversely correlate with the severity of CRSwNP [25].

However, contrary to our initial hypothesis, our results showed a direct correlation between MUC1 expression and the severity of CRSwNP. This finding could be explained by the close colocalization of MUC1 with CD15+ eosinophils (Figure 2). In fact, a recent study demonstrated that MUC1 promotes eosinophil survival through the release of its N-terminal domain, mediated by CCL4. This mechanism may contribute to the accumulation of eosinophils in the nasal mucosa, amplifying the inflammatory pathways underlying CRSwNP and explaining the paradox of our observation [2]. As a matter of fact, in Western Countries, CRSwNP is well known to be predominantly characterized by Type 2 inflammation with pronounced eosinophilia and the presence of high levels of Type 2 cytokines, such as IL-4, IL-5 and IL-13 [3]. Therefore, if MUC1 is involved in the regulation of eosinophil survival and promotes Type 2 inflammation, it could play a key role in the pathogenesis of CRSwNP. Since eosinophil is a widely recognized biomarker of disease severity, the correlation between MUC1 and eosinophilia suggests that MUC1 should also be considered a potential marker of CRSwNP severity [26].

Also, mast cells are now widely recognized as key players in the pathogenesis of the more severe forms of CRSwNP, contributing to chronic inflammation and tissue remodeling. Since previous oncological studies had shown a strong correlation between MUC1 expression and abundant mast cell infiltration in the context of renal and breast cancer, and considering the finding of increasing MUC1 in severe forms of CRSwNP, we expected to observe a colocalization of mast cells with MUC1 in nasal polyps as well [27,28]. Contrary to what was expected, both MUC1 and mast cells showed a close correlation with the severity of the disease, but not colocalization. Probably, in the context of the tumor microenvironment, among the different carcinogenic mechanisms, a sialylated glycoform associated with tumor-associated MUC1 could induce the differentiation of monocytes into tumor-associated macrophages (TAMs), which in turn become mast cells [29,30].

In CRSwNP, both MUC1 and mast cells appear to be independent factors of disease severity, although the common proportional increase with increasing CCG would suggest distinct but potentially complementary mechanisms in driving the disease’s progression. As is already known, most severe forms of CRSwNP are characterized by a mixed eosinophilic-mast cell inflammatory infiltrate and the two cytotypes appear to influence each other [31]. It is still unclear whether MUC1 plays a role in the interaction between eosinophils and mast cells or whether it acts independently, contributing separately to disease progression and influencing the underlying inflammatory mechanisms in CRS.

The main limitations of the study are the relatively small sample size and the analysis of MUC1, CD15, and tryptase expression exclusively through histological and immunofluorescence analyses, which provides valuable information on spatial distribution but does not allow precise quantification across the entire nasal mucosa. Future studies combining histological, molecular, and proteomic approaches (e.g., quantitative PCR, ELISA, or flow cytometry) are needed to better characterize expression levels and regulatory mechanisms.

Furthermore, larger-scale studies could be conducted to evaluate MUC-1 expression in Th1-endotype CRS patients to confirm these preliminary findings and further clarify the role of MUC1 and its immunological interactions in different CRS endotypes.

## 5. Conclusions

In conclusion, the role of MUC1 in the pathogenesis of CRSwNP represents an intriguing but still partially incomplete field of research. The correlation of MUC1 with the severity of CRSwNP, assessed according to CCG, offers a promising perspective for the use of MUC1 as a biomarker of CRSwNP. However, the variability in data regarding the function of MUC1, sometimes described as pro-inflammatory and other times as anti-inflammatory, raises important questions. This discrepancy highlights the need for further studies to clarify how different forms of MUC1 and its interactions with other immune cells may influence the inflammatory response in CRSwNP. Understanding these mechanisms underlying the interactions between MUC1, eosinophils and mast cells could have significant implications for the development of more targeted and personalized therapies.

Looking forward, it will be crucial to resolve the discrepancies regarding the different functions attributed to MUC1, as a deeper understanding of its biology could not only clarify its role in CRSwNP but also allow for the design of personalized therapeutic treatments. Furthermore, research should focus on identifying specific biomarkers that, along with MUC1, could improve early diagnosis and management of CRSwNP, thus reducing the risk of progression to more severe forms and improving the Quality of Life (QoL) for patients.

## Figures and Tables

**Table 1:** Patient demographics, comorbidities, nasal cytology, CCG, SNOT, and TNPS scores.

Patient	Sex	Age, Years	Surgical Procedures Underwent, n	Allergy	Asthma	ASA Sensitivity	Predominant Cytotype	CCG	SNOT-22	TNPS
1	M	66	1	No	No	No	Eos	Low (2)	24	5
2	M	59	1	No	No	No	Eos	Low (2)	8	6
3	M	31	1	No	No	No	Eos	Low (2)	20	5
4	M	48	1	No	No	No	Eos	Low (2)	17	5
5	M	63	1	No	No	No	Eos	Low (2)	22	6
6	M	71	1	No	No	No	Eos	Low (2)	22	7
7	M	55	1	Yes	No	No	Eos	Medium (5)	17	5
8	F	34	1	Yes	No	No	Eos	Medium (5)	27	6
9	M	63	2	Yes	No	No	Eos	Medium (5)	13	6
10	M	60	1	Yes	No	No	Eos	Medium (5)	8	7
11	M	40	1	Yes	No	No	Eos	Medium (5)	11	5
12	F	32	1	Yes	No	No	Eos	Medium (5)	27	5
13	M	54	1	Yes	Yes	No	Eos	High (7)	30	6
14	F	48	1	Yes	Yes	No	Eos- Mast cell	High (10)	23	7
15	F	30	2	Yes	Yes	Yes	Eos- Mast cell	High (10)	27	8
16	F	74	2	Yes	Yes	Yes	Eos- Mast cell	High (9)	16	5
17	M	33	2	Yes	Yes	No	Eos- Mast cell	High (9)	24	6
18	M	72	2	Yes	Yes	No	Eos- Mast cell	High (9)	42	8

## Data Availability

The data that support the findings of this study are available from the corresponding author, [R.G.], upon reasonable request.
